# Mefunidone Ameliorates Bleomycin-Induced Pulmonary Fibrosis in Mice

**DOI:** 10.3389/fphar.2021.713572

**Published:** 2021-09-24

**Authors:** Yuanyuan Han, Mao Jiang, Rongling He, Xin Lv, Xiaohua Liao, Yijun He, Fan Zhang, Lingzhi Long, Guoliang Jiang, Zhangzhe Peng, Lijian Tao, Gaoyun Hu, Jie Meng

**Affiliations:** ^1^ Department of Pulmonary and Critical Care Medicine, The Third Xiangya Hospital of Central South University, Changsha, China; ^2^ Hunan Key Laboratory of Organ Fibrosis, Changsha, China; ^3^ Department of Nephrology, Xiangya Hospital of Central South University, Changsha, China; ^4^ Department of Anesthesiology, Xiangya Hospital of Central South University, Changsha, China; ^5^ Department of Medicinal Chemistry, Xiangya School of Pharmaceutical Sciences, Central South University, Changsha, China

**Keywords:** mefunidone, pulmonary fibrosis, apoptosis, epithelial-mesenchymal transition, TGF-β

## Abstract

Idiopathic pulmonary fibrosis (IPF) is one of the most common and devastating interstitial lung diseases with poor prognosis. Currently, few effective drugs are available for IPF. Hence, we sought to explore the role of mefunidone (MFD), a newly synthesized drug developed by our team, in lung fibrosis. In this study, MFD was found to attenuate bleomycin (BLM) -induced lung fibrosis and inflammation in mice according to Ashcroft and alveolitis scoring. The protein contents and total cell counts in bronchoalveolar lavage fluids of BLM-treated mice were also lowered by MFD. Moreover, the elevation of TGF-β/Smad2 and phosphorylation of MAPK pathways was repressed by MFD. Additionally, MFD attenuated the swelling and vacuolization of mitochondria, lowered the ratio of apoptotic cells, restored the mitochondrial membrane potential, and reversed the expression of cleaved-caspase 3, Bcl-2 and Bax. Meanwhile, the level of epithelial marker, E-cadherin, was restored by MFD, while the levels of mesenchymal markers such as Snail and vimentin were down-regulated by MFD. Besides, MFD inhibited the expression of fibronectin and α-smooth muscle actin in TGF-β treated normal human lung fibroblasts. Thus, our findings suggested that MFD could ameliorate lung fibrosis, cell apoptosis and EMT potentially *via* suppression of TGF-β/Smad2 and MAPK pathways.

## Introduction

Idiopathic pulmonary fibrosis (IPF) is a progressive interstitial lung disease with increasing morbidity and mortality worldwide (Hutchinson et al., 2015). The etiology of IPF has not been fully understood, however, transforming growth factor β (TGF-β) has been largely reported to be essential in the initiation and development of IPF (Chanda et al., 2019). By directly binding to its receptors, TGF-β could phosphorylate Smad signaling pathway, and activate mitogen-activated protein kinase (MAPK) pathways (Ihn, 2008). Furthermore, strides have also been made in recent years that epithelial apoptosis, mitochondrial injury and epithelial-mesenchymal transition (EMT) are critically involved in the progression of lung fibrosis (Jablonski et al., 2017; Salton et al., 2019).

Apart from lung transplantation, only two drugs were approved by Food and Drug Administration (FDA) for the treatment of IPF, including pirfenidone (PFD) and nintedanib. Although previous studies have demonstrated that these two drugs could slow the decline of lung function (Somogyi et al., 2019), there is still no cure for IPF, which highlights the need for more effective therapies. Hence, fluorofenidone (AKFPD), a novel pyridone agent with similar chemical structure to PFD, was screened by our group. According to previous studies, AKFPD and PFD showed similar capacity in the aspects of anti-inflammation, anti-oxidation, and anti-fibrosis (Dai et al., 2020; Jiang et al., 2019; Liu. et al., 2015; Meng et al., 2012; Song et al., 2016; Tang et al., 2015; Zheng et al., 2018).

Based on AKFPD, we further improved its structure and found mefunidone (MFD). MFD [1-(4-((3-(4-methylpiperazin-1-yl) propyl)amino)benzyl)-5-(trifluoromethyl) pyridin-2(1H)-one], is a newly synthesized compound with higher water solubility and lower toxicity compared to AKFPD and PFD (Liu et al., 2015). According to previous reports, MFD inhibited renal inflammation and tubulointerstitial fibrosis in unilateral urinary obstructive kidney models at a considerably low dosage (Zhang et al., 2016). Besides, MFD could delay the progression of diabetic kidney disease, suppress the excessive accumulation of collagen and inhibit EMT (Jiang et al., 2021). Nevertheless, whether MFD is effective in lung fibrosis remains to be explored.

Hence, we aimed to investigate the effects of MFD on bleomycin (BLM)-induced lung fibrosis and elucidate its potential mechanism. Our data demonstrated that MFD could ameliorate BLM-induced pulmonary fibrosis, abate mitochondria-dependent cell apoptosis, and inhibit EMT via suppression of TGF-β/Smad2 and MAPK pathways.

## Methods and Materials

### Animal Studies

All the animal studies were approved by the Laboratory Animal Welfare Ethics Committee of Central South University. 8-week-old ICR mice were purchased from Silaike Company (Changsha, China), housed at Central South University, and given ad libitum access to food and water.

All the 40 mice were randomly divided into the following five groups with eight mice per group: saline group (Control group), BLM model group (BLM group), MFD-treated group (50 mg/kg/d) (MFD group), PFD-treated group (500 mg/kg/d) (PFD group), and AKFPD group (500 mg/kg/d) (AKFPD group). BLM (Nippon Kayaku, Japan) was solved in saline and given to mice via a single injection of tail vein at day 1 (10 mg/kg). MFD, PFD and AKFPD were solved in saline, 0.5% CMCNa, and 0.5% CMCNa respectively, and administered daily by gavage for 28 days. All the mice were sacrificed at day 28. Lung tissues and bronchoalveolar lavage fluids (BALFs) were collected and stored for subsequent experiments.

### Histopathology and Immunohistochemistry

Lungs were perfused *in situ* with cold saline before removal. The right lungs were snap-frozen in liquid nitrogen. The left lungs were fixed in 10% formaline buffer, embedded in paraffin, and sectioned at 4 µm. Sections were fully deparaffinized and rehydrated. Hematoxylin and Eosin (H and E) staining and Masson’s trichrome staining were performed following the manufacturers’ protocols. Alveolitis score (Szapiel et al., 1979) and Ashcroft score (Ashcroft et al., 1988) were respectively applied to assess the inflammation and fibrosis of the lungs.

For immunohistochemistry, the sections were rehydrated via gradient ethanol, treated with unmasking solution for antigen retrieval, and incubated with primary antibodies at 4°C overnight. Primary antibodies used included: fibronectin (Fn) (Proteintech, USA), α-smooth muscle actin (α-SMA) (Abcam, USA) and collagen Ⅰ (Abcam, USA). Slides were further stained with secondary antibodies and DAB the following day. Images were obtained at Central South University on a Nikon microscope, and quantified using Image-Pro Plus 7.0.

### Cell Culture and Treatment

The murine alveolar epithelial type Ⅱ cells (MLE-12) were obtained from Zhongqiao Xinzhou Biotechnology Company (Shanghai, China). Normal human lung fibroblasts (NHLF) were primarily cultured from normal human lungs as previously reported (Liu. et al., 2015). Cells were grown in DMEM/F12 or DMEM medium (Gibco, USA) supplemented with 10% FBS (Gibco, USA) and 1% Penicillin-Streptomycin Liquid (Gibco, USA), and maintained at 37°C with 5% CO_2_. When the cells grow to 70–80% of confluence, they were incubated with serum-free medium overnight. TGF-β (Peprotech, USA), BLM (Yeshen, China) and MFD were filtered with 0.22 µm filters before use. Each experiment was replicated for at least three times.

### Transmission Electron Microscope

Transmission electron microscope was used to observe the morphology of mitochondria. Right lungs were collected and immediately fixed in 2.5% glutaraldehyde, dehydrated with ethanol and acetone, embedded in resin, and cut into sections of ∼60 nm. Images were acquired on a Leica TEM at Central South University.

### A Terminal Deoxynucleotidyl Transferase Mediated Nick End Labeling Assay

A terminal deoxynucleotidyl transferase mediated nick end labeling (TUNEL) kit (Roche, Switzerland) was used to detect cell apoptosis. Lung sections were fully deparaffined and rehydrated, permeablized with protease K, incubated with TUNEL reaction solution followed by DAB solution. Images were acquired on a fluorescent microscope.

### Cell Counts, Protein and TGF-β Level Detection in BALFs

BALFs were collected by intratracheal instillation of 3 ml of sterile saline solution with gentle aspiration. The recovery of the lavage fluids ranged from 2 to 2.6 ml. Total cell numbers in BALFs were counted using hemocytometer with 0.4% trypan blue (Sigma, USA). The protein in BALFs was measured using the BCA Protein Assay Kit (Thermo Fisher Scientific, USA). The TGF-β level in BALFs was detected using a TGF-β enzyme-linked immunosorbent assay kit (Invitrogen, USA). The assay was performed following the manufacturer’s protocol. The results were presented as the mean of duplicates in pictograms per ml.

### Western Blotting

Whole proteins from lung tissues or cells were extracted using RIPA buffer containing protease inhibitors. Collected lysates were boiled at 95°C for 5 min, separated on 8–12% SDS-PAGE gels, and transferred to PVDF membranes. Membranes were blocked and then incubated overnight at 4°C with primary antibodies from the following sources: Fn (Abcam, USA), α-SMA (Sigma, USA), collagen-Ⅰ (Abcam, USA), Bax (CST, USA), Bcl-2 (CST, USA), cleaved-caspase 3 (Proteintech, USA), E-cadherin (Proteintech, USA), Snail (Proteintech, USA), vimentin (Proteintech, USA), *p*-ERK1/2 (CST, USA), ERK1/2 (CST, USA), *p*-Smad2 (CST, USA), α-tubulin (Proteintech, USA), GAPDH (Proteintech, USA), and TGF-β (Abcam, USA). The bands were visualized with ECL detection reagents (Thermo Fisher Scientific, USA) and quantified using ImageJ software.

### RNA Extraction and Real-Time PCR Quantitation

Total RNA was extracted from lung tissues with Trizol reagent (Thermo Fisher Scientific, United States ) according to the manufacturer’s instructions. Reverse transcription reactions were carried out with 1 μg of total RNA using a cDNA synthesis kit (Thermo Fisher Scientific, United States ). The synthesized cDNA was further used for PCR where β-actin was used as loading control. SYBR Green gene expression assay (Thermo Fisher Scientific, United States ) was performed in a CFX96 Real-Time System (Bio-Rad Laboratories, United States ) to measure the mRNA expression. The specific primers were designed from the GenBank sequences and synthesized by Sangon Biotec (Shanghai, China). The forward primer of β-actin was 5′-GGC​CAA​CCG​TGA​AAA​GAT​GA-3′, and the reverse primer was 5′-GAC​CAG​AGG​CAT​ACA​GGG​ACA​A-3’. The forward primer of TGF-β was 5′-GCA​ACA​TGT​GGA​ACT​CTA​CCA​GA-3′, and the reverse primer was 5′-GAC​GTC​AAA​AGA​CAG​CCA​CTC​A-3’.

### Cell Counting Kit-8 Assay

A cell counting kit-8 (CCK-8) was purchased from APExBIO Technology LLC (Houston, USA) to detect the effects of MFD on the viability of MLE-12 cells. It was performed following the manufacturer’s protocol. An aliquot of 100 µl of cell suspension (about 3,500 cells/well) was inoculated in each well of the 96-well plate, and pre-incubated in a humidified incubator for 24 h. After that, cells were treated with different concentrations of MFD from 0 to 320 μg/ml for 24 h or 48 h, followed by incubation with CCK-8 solution for 2 h at 37°C. The absorbance of each well was detected at 450 nm using a microplate reader. The number of living cells was directly proportional to the absorbance.

### Flow Cytometry for Detection of Cell Apoptosis

MLE-12 cells were inoculated in each well of 6-well plates with DMEM/F12 medium containing 10% fetal bovine serum (FBS) for 24 h. Each plate contained three groups: control, BLM, and BLM plus MFD group. After incubation in medium with 2% FBS for 12 h, MFD group was pretreated with MFD (40 μg/ml) for 2 h. Afterwards, BLM group and MFD group were incubated with BLM (400 μg/ml) for 24 h to induce cell death. Cells were then collected, and stained with the FITC Annexin V and PI staining kit (BD Pharmingen, USA) according to the manufacturer’s instructions. This assay discriminates between intact (FITC^−^/PI^−^), early apoptotic (FITC^+^/PI^−^), and later apoptotic (FITC^+^/PI^+^) cells. Data were obtained with an Aurora-10 (Cytek) flow cytometer and analyzed with FlowJo 10.1.

### Mitochondrial Membrane Potential Measurement

A TMRM assay kit (Thermo, USA) was used to measure mitochondrial membrane potential (MMP). It was performed following the manufacturer’s protocol. Briefly, MLE12 cells were seeded in 6-well plates with DMEM/F12 medium for 24 h. Each plate contained three groups: control, BLM, and BLM plus MFD group. After incubation in medium with 2% FBS for 12 h, MFD group was pretreated with MFD (40 μg/ml) for 2 h. Afterwards, both BLM group and MFD group were incubated with BLM (400 μg/ml) for 24 h to induce cell death. Diluted TMRM reagent was added, incubated for 30 min, and then exchanged with live cell imaging buffer. TMRM was detected at 575 nm. Data were acquired with an Aurora-10 (Cytek) flow cytometer.

### Statistical Analysis

All data were expressed as the mean ± SD. All experiments were conducted with more than three independent replications. *p* values were calculated through one-way analysis of variance. *p* < 0.05 was considered significantly different. All analyses were performed using SPSS 22.0 and GraphPad Prism 8.0.

## Results

### Mefunidone Ameliorated BLM-Induced Pulmonary Fibrosis and Inflammatory Injuries

**FIGURE 1:**
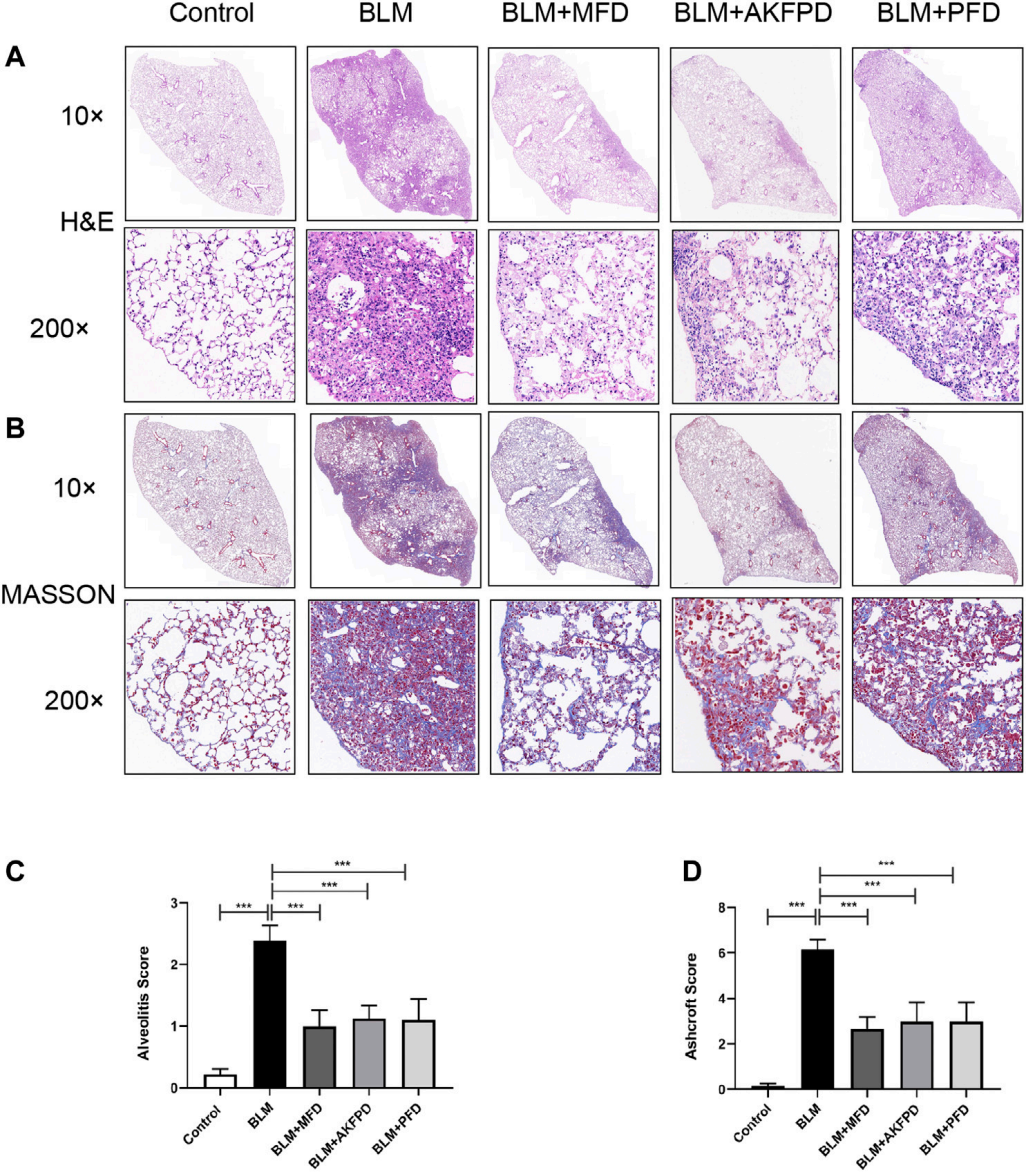
Mefunidone ameliorated bleomycin-induced pulmonary fibrosis. Pulmonary fibrosis was induced by bleomycin (10 mg/kg, iv) and the lungs were collected 28 days after bleomycin injection. Mice in BLM + MFD, BLM + AKFPD and BLM + PFD groups were treated with mefunidone, fluorofenidone or pirfenidone respectively by gavage for 28 days **(A–B)**: Representative images of H and E staining **(A)** and Masson’s trichrome staining **(B)** of lung tissues from different groups of mice **(C-D)**: Alveolitis scoring **(C)** and Ashcroft scoring **(D)** of different groups. All data were presented as mean ± SD. ANOVA was used for comparison in **(C-D)**. ***p < 0.001.

**FIGURE 2:**
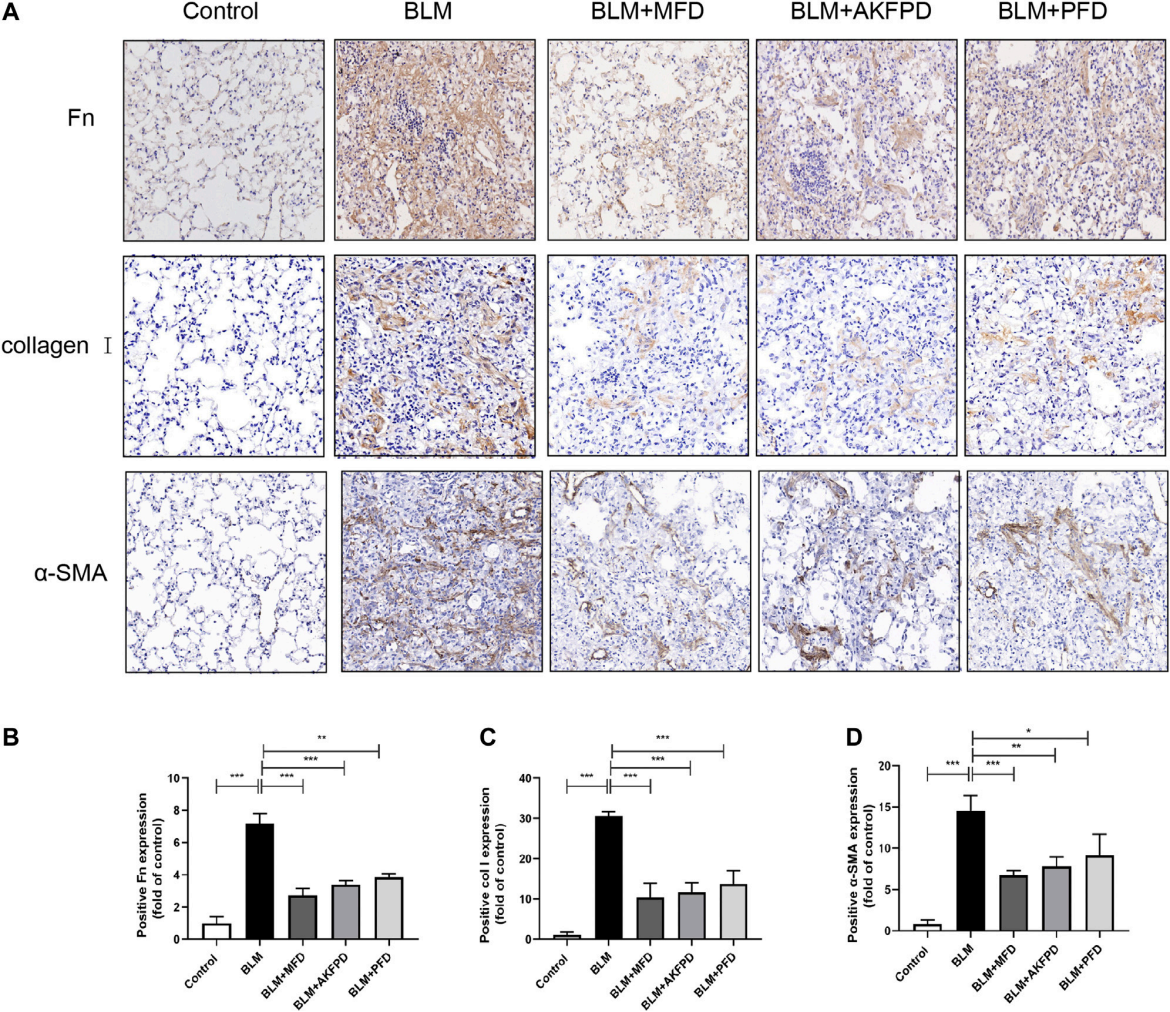
Mefunidone inhibited the deposition of extracellular matrix. **(A–D)**: Representative images **(A)** and quantitative analysis of immunohistochemistry for fibronectin **(B)**, collagen Ⅰ **(C)** and α-SMA **(D)** in lung tissues from different groups of mice. The magnification in A was ×200. All data were presented as mean ± SD. ANOVA was used for comparison in **(B–D)**.*p < 0.05; **p < 0.01; ***p < 0.001.

**FIGURE 3:**
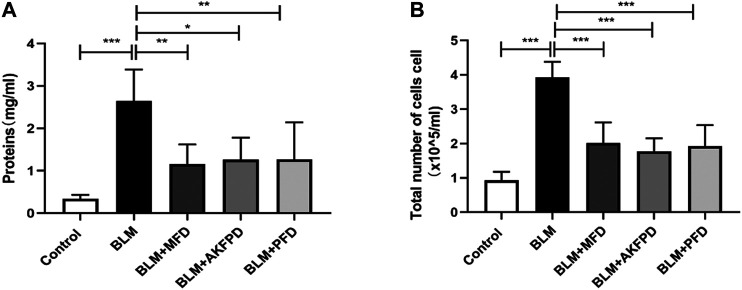
Mefunidone decreased protein concentration and cell count in bronchoalveolar lavage fluids **(A–B)**: Total protein concentrations **(A)** and cell counts **(B)** in bronchoalveolar lavage fluids of mice. All data were presented as mean ± SD. ANOVA was used for comparison in **(A–B)**.*p < 0.05; **p < 0.01; ***p < 0.001.

BLM-induced lung fibrosis in mice has been the most widely used experimental model of IPF. At day 28, H&E staining (Figure 1A) and Masson’s trichrome staining (Figure 1B) demonstrated extensive distortion of lung tissues, massive infiltration of leukocytes and excessive collagen deposition in BLM-administered mice as compared to saline-treated mice. When compared to BLM group, MFD showed much restoration from BLM-induced injuries, similar to AKFPD or PFD (Figure 1A,B). Meanwhile, the alveolitis score (Figure 1C) and Ashcroft score (Figure 1D), which reflect the severity of inflammation and fibrosis respectively, were significantly reduced by MFD, AKFPD and PFD treatment. Additionally, immunohistochemical staining of Fn, α-SMA, and collagen Ⅰ showed notable increases in mice treated with BLM, which was significantly attenuated by MFD, PFD and AKFPD (Figures 2A–D). No significant difference was observed among MFD, PFD and AKFPD groups, but the dosage of MFD (50 mg/kg/d) was 80% lower than PFD (500 mg/kg/d) and AKFPD (500 mg/kg/d). Furthermore, we collected the BALFs of mice, and found that MFD could reduce BLM-induced elevation of inflammatory cell counts (Figure 3A) and protein contents (Figure 3B). Taken together, these evidence suggested that MFD could ameliorate BLM-induced pulmonary fibrosis and inflammatory injuries with considerably low dosage.

### Mefunidone Suppressed TGF-β/Smad and Mitogen-Activated Protein Kinase Pathways

**FIGURE 4:**
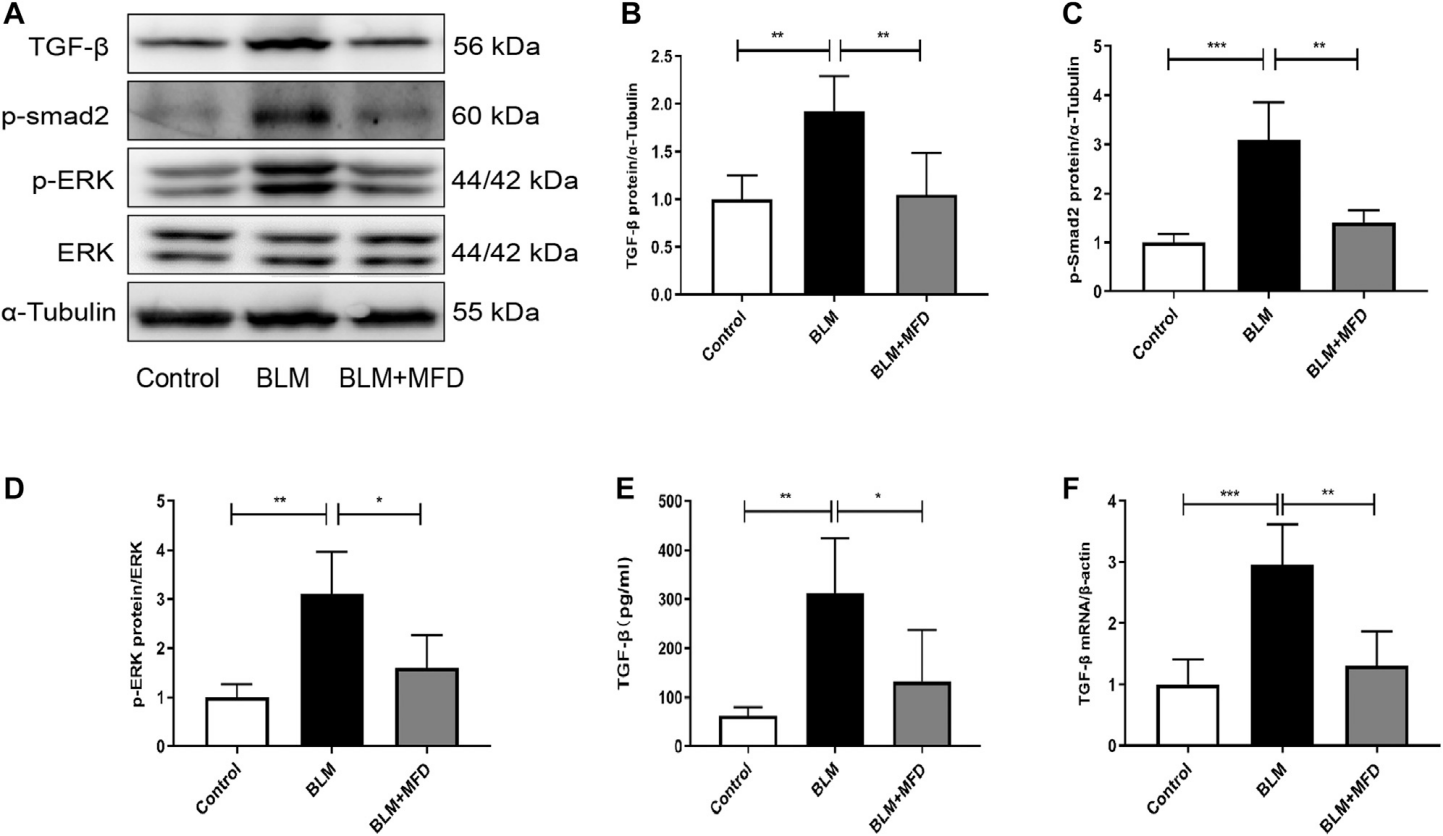
Mefunidone suppressed TGF-β/Smad2 and MAPK pathways **(A-D)**: Western blotting **(A)** and quantitative analysis of TGF-β **(B)**, *p*-Smad2 **(C)** and *p*-ERK **(D)** in mice lung tissues **(E)**: The TGF-β concentration in BALFs of mice **(F)**: The mRNA levels of TGF-β in lung tissues of mice. All data were presented as mean ± SD. ANOVA was used for comparison in **(B-D).** *p < 0.05; **p < 0.01; ***p < 0.001.

As one of the most important features in the initiation and progression of lung fibrosis, the up-regulation of TGF-β has been largely reported. In our study, an increase of TGF-β level in BALF was also observed after BLM treatment (Figure 4E). We further confirmed the elevation of TGF-β in lung tissues via qRT-PCR (Figure 4F) and western blotting (Figure 4A), while treatment of MFD significantly diminished the elevation of TGF-β. Smad proteins and MAPK pathways are key components in the TGF-β signaling pathway. Hence, we further detected the levels of related proteins. As shown in Figures 4A–D, BLM-administered mice showed elevation of phosphorylation of Smad2 and ERK1/2, which were diminished by MFD. In summary, these findings indicated that MFD could down-regulate TGF-β/Smad2 and MAPK pathways.

### Mefunidone Attenuated BLM-Induced Cell Apoptosis and Mitochondrial Injury *in vivo*

**FIGURE 5:**
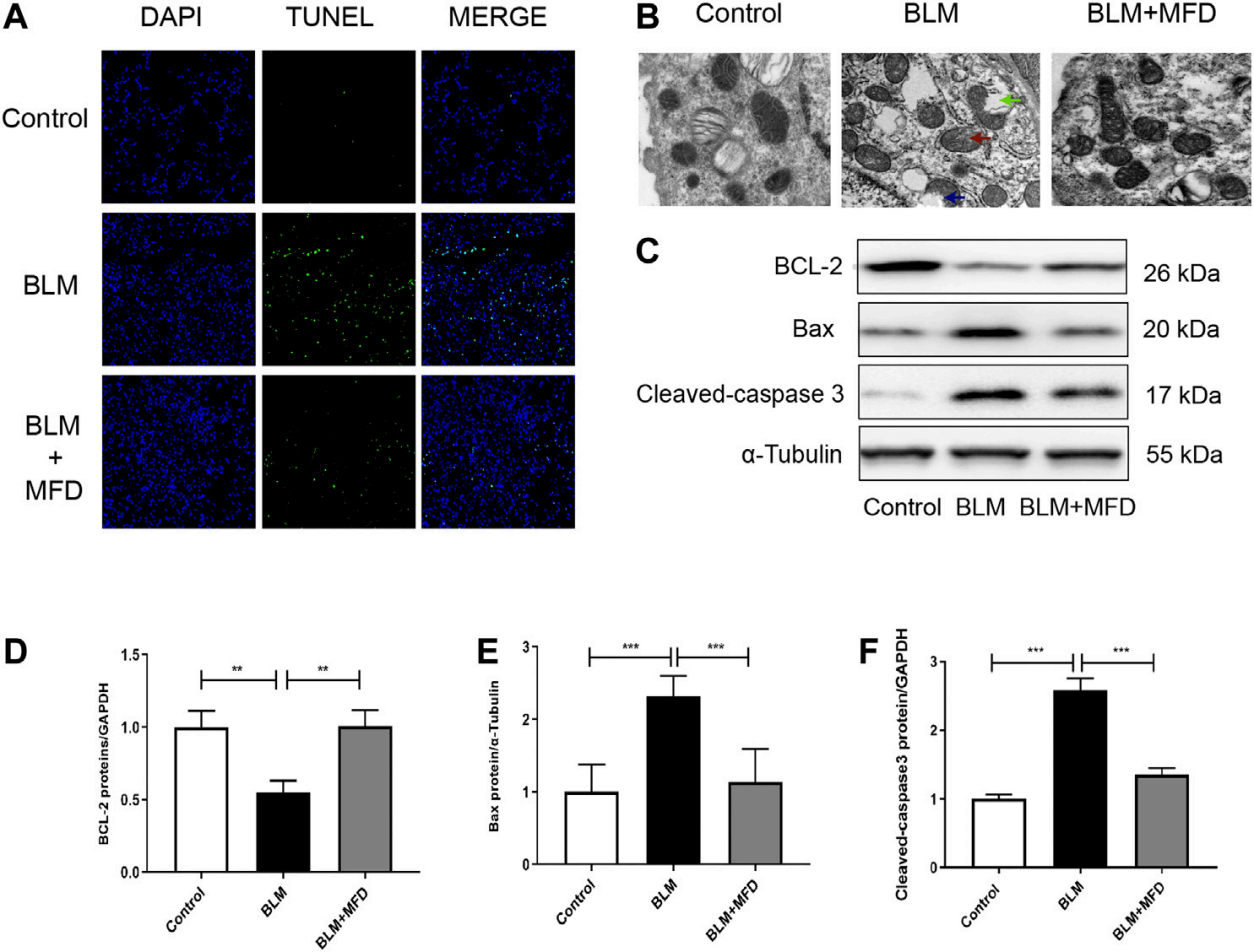
Mefunidone attenuated bleomycin-induced mitochondrial injury and cell apoptosis **(A)**: Representative images of TUNEL assay of lung tissues from different groups. DAPI stained the nuclei, and TUNEL stained the apoptotic cells. The magnification was ×200 **(B)**: Representative images of mitochondrial morphology from different groups captured by transmission electron microscope. The treatment of bleomycin (BLM) induced mitochondrial vacuolization (see the blue arrow), swelling (see the red arrow) and rupture of outer membrane (see the green arrow). The magnification was ×20,000 **(C–F)**: Western blotting **(C)** and quantitative analysis of Bcl-2 **(D)**, Bax **(E)** and Cleaved-caspase 3 **(F)** in lung tissues from different groups. All data were presented as mean ± SD. ANOVA was used for comparison in **(D-F)**. *p < 0.05; **p < 0.01; ***p < 0.001.

Previous studies have demonstrated that cell apoptosis plays an important role in the pathogenesis of IPF (Jablonski et al., 2017). In our study, more TUNEL positive cells were observed in the BLM-administered lungs compared to saline ones, which was remarkably reduced by treatment of MFD (Figure 5A). Additionally, when transmission electron microscope was used to observe the morphology of mitochondria, we found that BLM induced the vacuolization, swelling and rupture of the outer membrane of mitochondria in type Ⅱ alveolar epithelial cells (ACE Ⅱ), which was reversed by MFD (Figure 5B). The improvement of mitochondrial injury supported the possibility that MFD might suppress mitochondria-associated cell apoptosis. To testify this, we measured the levels of proteins associated with mitochondrial apoptosis, including cleaved-caspase 3, Bax and Bcl-2. According to the western blotting analysis, BLM induced the increase of cleaved-caspase three and Bax, which were suppressed by MFD. Moreover, MFD restored the expression of Bcl-2 (Figures 5C–F). Taken together, these results supported that MFD could attenuate mitochondrial-associated cell apoptosis.

### Mefunidone Abated Lung Epithelial Cell Apoptosis

**FIGURE 6:**
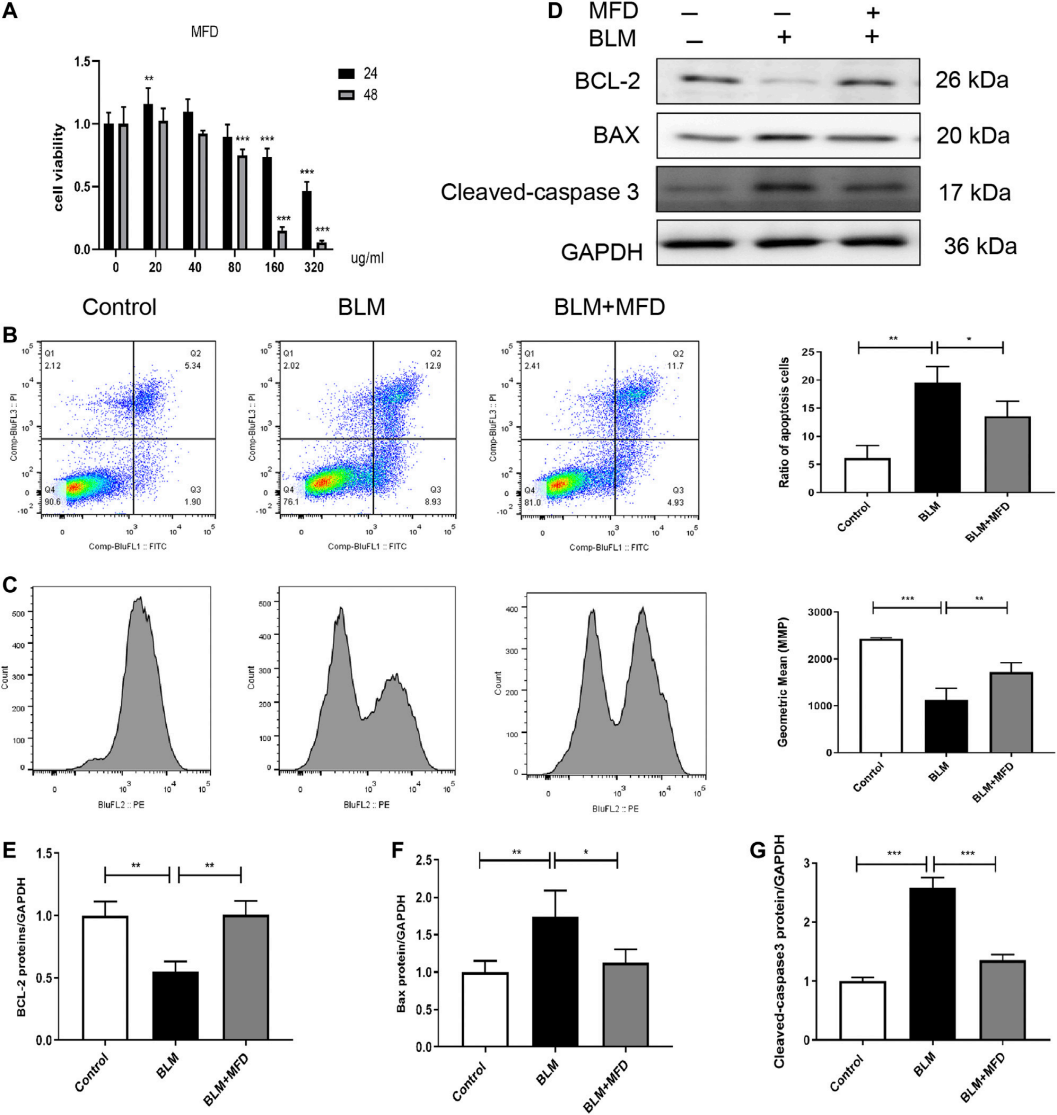
Mefunidone abated belomycin-induced apoptosis of MLE-12 cells. **(A)**: The effect of different concentration and pretreatment duration of mefunidone on viability of MLE-12 cells detected by CCK-8 assay. MLE-12 cells were treated with mefunidone at concentrations of 0, 20, 40, 80, 160 and 320 μg/ml for 24 h or 48 h. **(B)**: Flow cytometry of FITC/PI and quantitative comparison of MLE-12 cells treated with bleomycin (400 μg/ml) or PBS with/without mefunidone (40 μg/ml) pretreatment. **(C)**: Mitochondrial membrane potential and quantitative comparison of MLE-12 cells treated with bleomycin (400 μg/ml) or PBS with/without mefunidone (40 μg/ml) pretreatment **(D–G)**: Western blotting **(D)** and quantitative analysis of Bcl-2 **(E)**, Bax **(F)**, and Cleaved-caspase 3 **(G)** in MLE-12 cells treated with bleomycin (400 μg/ml) with/without MFD (40 μg/ml) pretreatment. All experiments were conducted with more than three independent replications. Data were presented as mean ± SD. ANOVA was used for comparison used in **(B,C, and E–G)**. *p < 0.05; **p < 0.01; ***p < 0.001.

We next investigated the effect of MFD on cell apoptosis *in vitro*. We adopted MLE-12 cells, a cell line of mouse type Ⅱ alveolar epithelial cell, and firstly tested the effects of different concentration and time duration of MFD on the viability of MLE-12 cells. According to the results of CCK-8 assay (Figure 6A), we decided to pretreat the MLE-12 cells with 40 μg/ml of MFD for 24 h or 48 h for subsequent experiments.

Primarily, flow cytometry was used to detect the apoptosis of MLE-12 cells. As shown in Figure 6B, more FITC^+^/PI^+^ cells, suggesting more apoptotic cells, were observed in the BLM-administered MLE-12 cells. Whereas, the treatment of MFD reduced the ratio of apoptotic cells. Secondly, to investigate whether the apoptosis was mitochondria-dependent, we measured the alteration of mitochondrial membrane potential (MMP). As shown in Figure 6C, BLM-induced loss of MMP was restored by MFD treatment. Meanwhile, we further tested the levels of proteins related to mitochondria-associated cell apoptosis. MFD could significantly decrease cleaved-caspase three and Bax, and increase Bcl-2 (Figures 6D–G), which was consistent with the results *in vivo*. In summary, MFD could suppress the apoptosis of MLE-12 cells, potentially through a mitochondria-dependent way.

### Mefunidone Inhibited Epithelial-To-Mesenchymal Transition Both *in vivo* and *in vitro*

**FIGURE 7:**
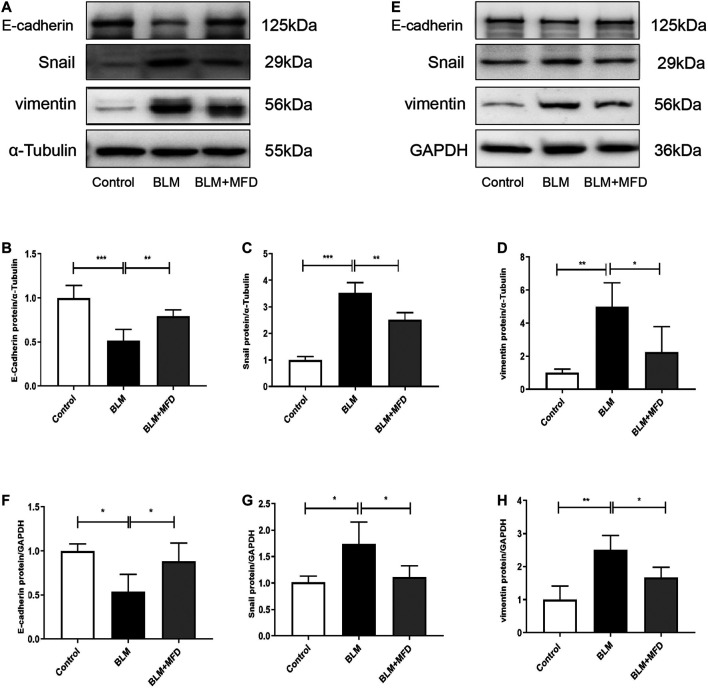
Mefunidone inhibited epithelial-to-mesenchymal transition both *in vivo* and *in vitro* **(A–D)**: Western blotting **(A)** and quantitative analysis of E-cadherin **(B)**, Snail **(C)** and vimentin **(D)** in lung tissues from different groups **(E–H)**: Western blotting **(E)** and quantitative analysis of E-cadherin **(F)**, Snail **(G)** and vimentin **(H)** in MLE-12 cells treated with bleomycin (400 μg/ml) with/without mefunidone (40 μg/ml) pretreatment. All experiments were conducted with more than three independent replications. Data were presented as mean ± SD. ANOVA was used for comparison in **(B–D)** and **(F–H)**. *p < 0.05; **p < 0.01; ***p < 0.001.

EMT is a pathophysiology process where epithelial cells lose part of their features, while gaining mesenchymal characteristics. Although the pathogenesis of IPF has not been fully understood, EMT has been largely reported to be involved (Han et al., 2015). As shown in Figures 7A–D, MFD restored the expression of E-cadherin (an epithelial marker), and reduced that of Snail (which promotes EMT at transcriptional level), and vimentin (a mensenchymal marker). Further, we investigated the effects of MFD on EMT *in vitro*. The levels of E-cadherin, Snail and vimentin in BLM-treated MLE-12 cells were tested using western blotting. Consistent with what found *in vivo*, MFD significantly increased the expression of E-cadherin, and decreased that of Snail and vimentin (Figures 7E–H). Collectively, our findings demonstrated that MFD inhibited EMT both *in vivo* and *in vitro*.

### Mefunidone Inhibited the Formation of Extracellular Matrix *in vitro*

**FIGURE 8:**
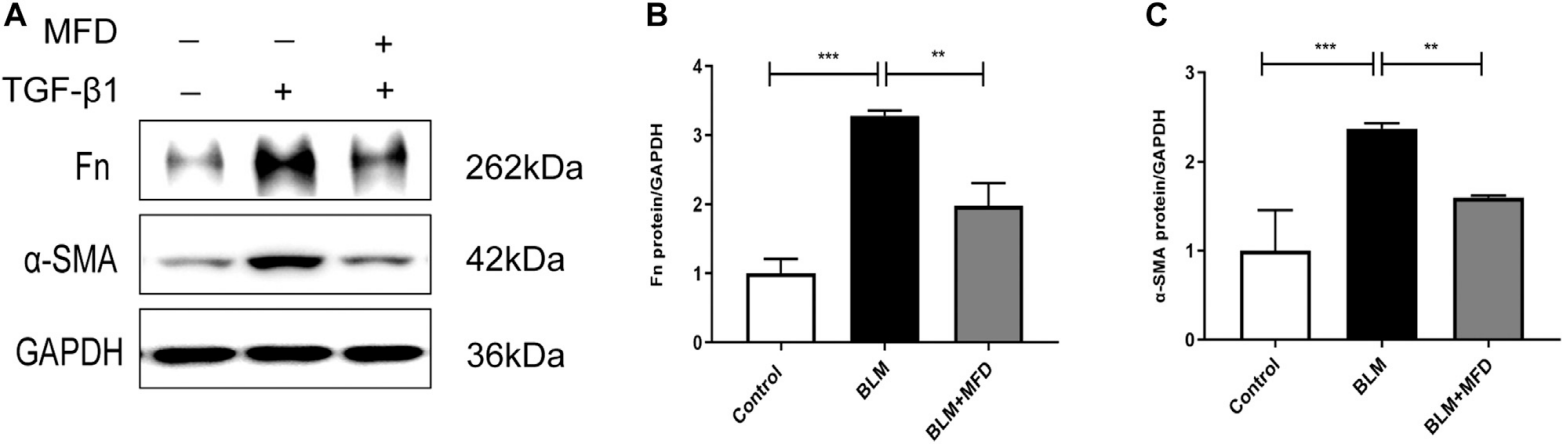
Mefunidone inhibited the formation of extracellular matrix in fibroblast **(A–C)**: Western blots **(A)** and quantitative analysis of fibronectin **(B)** and α-SMA **(C)** in normal human lung fibroblasts (NHLF) treated with TGF-β1 (10 ng/ml) with/without mefunidone (40 μg/ml) pretreatment. All experiments were conducted with more than three independent replication. Data were presented as mean ± SD. ANOVA was used for comparison in **(B–C)**. **p < 0.01; ***p < 0.001.

As reported, excessive accumulation of extracellular matrix is intrinsically linked to the development of lung fibrosis (Chanda et al., 2019). To further confirm the anti-fibrotic role of MFD *in vitro*, we selected normal human lung fibroblasts (NHLFs) to testify that. According to Figures 8A–C, the expressions of Fn and α-SMA were significantly reduced by MFD.

## Discussion

IPF is a devastating disease with poor prognosis which requires more effective therapies. Currently, there are only two drugs available for IPF, including PFD and nintedanib. AKFPD and MFD are newly synthesized and orally available compounds that are developed by our team. Our previous data indicated that AKFPD and MFD inhibited renal interstitial fibrosis and collagen accumulation in rat/murine models of unilateral ureteral obstruction and diabetic kidney diseases. Still, no data was available concerning the effects of MFD on lung fibrosis. The present study demonstrated that MFD could ameliorate BLM-induced pulmonary fibrosis and inflammatory injuries in mice. Meanwhile, EMT and mitochondria-associated cell apoptosis were suppressed by MFD. Furthermore, MFD might decrease TGF-β levels and phosphorylation of Smad2 and ERK1/2. Collectively, these findings suggest the possibility that MFD might attenuate pulmonary fibrosis, inhibit cell apoptosis, and suppress EMT via down-regulation of TGF-β/Smad2 and MAPK pathways.

BLM has been extensively used as an agent for lung fibrosis model in mice. In this study, we conducted a single injection of BLM via tail vein, and found massive fibrosis and inflammation in the lung at day 28. The treatment of MFD strongly inhibited lung fibrosis and collagen accumulation according to histopathology scoring and IHC staining. Meanwhile, MFD inhibited the infiltration of inflammatory cells in airways and lowered protein concentration in BALFs. Thus, these evidence supported that MFD could exert anti-fibrotic effects and ameliorate BLM-induced inflammation, which is in agreement with MFD’s anti-fibrotic role in renal fibrosis (Liu et al., 2015; Zhang et al., 2016; Jiang et al., 2021). Notably, no significant difference was observed concerning the anti-fibrotic effects of PFD, AKFPD and MFD, despite the dosage of MFD was 80% lower.

As is previously reported, cell apoptosis is one of the hallmarks of senescent phenotype present in fibrotic lungs (Jablonski et al., 2017). Mitochondrion is particularly involved in the apoptotic process and considered to act as a central coordinator of cell death. Dysfunction of mitochondrion could induce dissipation of MMP, alter the permeability of mitochondrial outer membrane, and release apoptogenic proteins (Li et al., 2012), which is triggered by the oligomerization of Bax in the mitochondrial outer membrane to form transition pores (Roufayel and Kadry, 2017). Bcl-2, however, could exert anti-apoptotic effects by inhibiting Bax activation (Roufayel, 2016). In this study, we provide several lines of evidence that implicate the role of MFD in inhibiting cell apoptosis. Firstly, the swelling and vacuolization of mitochondria was attenuated by MFD according to TEM scan. Meanwhile, MFD reversed the BLM-induced alteration of MMP, and the levels of cleaved-caspase 3, Bax and Bcl-2 both *in vivo* and *in vitro*, suggesting that MFD inhibited cell apoptosis potentially via a mitochondria-dependent way.

There is abundant evidence that EMT plays a vital role in the development of IPF (Yamada et al., 2008; Jolly et al., 2018; Gao et al., 2020). EMT is an evolutionarily conserved process in which epithelial cells lose contact adhesion, and acquire some mesenchymal features of migration and production of ECM (Y. Xu et al., 2020). Snail is a transcription factor which could repress the expression of E-cadherin, and induce the expression of mesenchymal proteins such as vimentin, α-SMA and metalloproteinases (Wang et al., 2013; Wang et al., 2021). In this study, we found that MFD could restore the expression of E-cadherin and reduce that of Snail and vimentin both *in vivo* and *in vitro*, namely inhibit EMT.

Although the mechanism underlying MFD’s anti-fibrotic role has not been comprehensively determined, we identified several possibilities. TGF-β is one of the key signal cascades in the pathogenesis of IPF which might work as a master switch to trigger sequential interconnected events (Bartram and Speer, 2004; Fernandez and Eickelberg, 2012; Chanda et al., 2019). Convincing evidence have demonstrated that TGF-β facilitates epithelial cells to undergo a phenotype shift characterized by adoption of mesenchymal features (Ding et al., 2017; Su et al., 2020). Moreover, TGF-β could potentially induce the apoptosis of alveolar epithelial cells (Siegel and Massague, 2003). Meanwhile, TGF-β signaling plays a key role in mediating myofibroblast activation and subsequent production of collagen and other ECM components (Xu et al., 2003). In our study, we found that TGF-β was elevated in the setting of BLM-induced lung fibrosis, and MFD significantly reduced the levels of TGF-β in the BALFs and lung tissues. Smad2 is known as one of the canonical downstream of TGF-β signaling pathway (Massague and Chen, 2000). Consistently, the phosphorylation of Smad2 was inhibited by MFD. In addition, as one of the noncanonical TGF-β pathways, MAPKs are consumed to be involved in lung fibrosis (Ihn, 2008; J.; Meng et al., 2012). As was reported, the activation of ERK/JNK/p38 could promote the synthesis of collagen (Meng et al., 2014; Goda et al., 2020). In this study, the treatment of MFD suppressed the phosphorylation of MAPKs. Taken together, these data suggested that the anti-fibrotic role of MFD might at least partly depend on the suppression of TGF-β/Smad and MAPK pathways.

To our knowledge, this is the first study to investigate the role of MFD in lung fibrosis. In summary, we demonstrated that MFD could attenuate BLM-induced lung fibrosis, reduce cell apoptosis and EMT, and suppress TGF-β/Smad2 and phosphorylation of MAPK pathway. Therefore, considering its high solubility and comparatively lower toxicity, MFD, as a multi-target compound, might be a promising candidate for pulmonary fibrosis.

## Data Availability

The original contribution presented in the study are included in the article/Supplementary Material, further inquiries can be directed to the corresponding author.
